# Supplementary material for: Social determinants of pain, distress, and quality of life in injured workers: A cross-sectional and longitudinal analysis of patient-reported outcomes
Source: PLoS One. 2026 Apr 24;21(4):e0346697. doi: 10.1371/journal.pone.0346697 (PMC13108777; doi:10.1371/journal.pone.0346697)
Supplement: S1 Table — Respondents were free to select as many categories as applies to them. (DOCX) [file pone.0346697.s001.docx]

| **Racial/Ethnic Category** | **Frequency (%)** |
| --- | --- |
| Black (e.g., African, African Canadian, Afro-Caribbean descent) | 11 (5.6%) |
| East Asian (e.g., Chinese, Japanese, Korean, Taiwanese descent) | 10 (5.1%) |
| Indigenous (First Nations, Inuk/Inuit, Metis descent) | 7 (3.5%) |
| Latin American (e.g., Hispanic or Latin American descent) | 8 (4.0%) |
| Middle Eastern (e.g., Arab, Persian, West Asian (e.g., Afghan, Egyptian, Iranian, Kurdish, Lebanese, Turkish) descent) | 5 (2.5%) |
| South Asian (e.g., Bangladeshi, Indian, Indo-Caribbean, Pakistani, Sri Lankan descent) | 18 (9.1%) |
| Pacific Islander (e.g., Native Hawaiian, Samoan, Guamanian/Chamorro, Fijian, Maori, or Tongan descent) | 1 (0.5%) |
| White (e.g., European descent) | 124 (62.6%) |
| Race Category not listed (optional: Specify) | 6 (3.3%) |
| Do not know or I prefer not to disclose | 8 (4.4%) |
