# Supplementary material for: Social determinants of pain, distress, and quality of life in injured workers: A cross-sectional and longitudinal analysis of patient-reported outcomes
Source: PLoS One. 2026 Apr 24;21(4):e0346697. doi: 10.1371/journal.pone.0346697 (PMC13108777; doi:10.1371/journal.pone.0346697)
Supplement: Attachment — Submitted filename: Response to Reviewers.docx [file pone.0346697.s003.docx]

**Response to Reviewer Comments**

We thank the Editor and reviewers for their time reviewing this manuscript. We are encouraged by the very positive comments, and have found the suggestions for improvement to have strengthened the paper further. Below we have copy/pasted the verbatim comments from the reviewers, and provided a point-by-point response to each one. We look forward to the results of the next round of peer review.

**Reviewer #1**: This manuscript presents a well-designed and methodologically rigorous study investigating the prognostic utility of three commonly used clinical self-report tools (NPRS, TIDS, and EQ-5D-5L) in adults with acute musculoskeletal (MSK) injuries, with a focus on potential differences in functioning across sociodemographic subgroups. Overall, the study is technically sound, and the data support the conclusions drawn.

Strengths:

Novelty and Significance: The study addresses an important gap in prognostic research by examining how social and demographic factors may influence both baseline scores and predictive accuracy of clinical tools, which has implications for personalized rehabilitation. The inclusion of both self-reported and administrative recovery outcomes adds depth and robustness to the findings.

Methodology: The study uses validated instruments, clearly defined outcomes, and appropriate statistical analyses, including AUC analyses for discriminative accuracy. Data cleaning and handling of missing responses are described transparently.

Clarity of Reporting: The manuscript is well-organized, with clear presentation of results in tables and figures. Discussion thoughtfully interprets the findings in light of previous literature, acknowledges limitations, and proposes directions for future research.

Ethical and Research Integrity: There are no apparent concerns regarding dual publication, research ethics, or data integrity. Consent procedures and data handling are appropriately described.

Areas for Consideration / Minor Suggestions:

While sociodemographic variables were dichotomized for analysis, further discussion of potential limitations of broad categorizations (e.g., “non-white”) is included in the manuscript. Future work could explore finer-grained categories with larger samples.
**RESPONSE: We note that reviewer 3 has also highlighted this issue, and it is forefront in our own minds. We’ve added a considerable section to the relevant paragraph of the Discussion (paragraph 4, lines 388 – 401) to make this issue explicit.**

The sample size for some subgroup analyses is relatively small, which may limit the power to detect differences. The authors acknowledge this and interpret findings cautiously.
**RESPONSE: We’re pleased that the reviewer has also noted our call for caution when interpreting some of the results, particularly those with smaller-than-desired numbers in some cells.**

The manuscript could benefit from a brief note on the clinical implications of the findings, particularly regarding how clinicians might use TIDS or EQ-5D-5L scores in practice to inform individualized prognostic assessments.

**RESPONSE: We’ve attempted to highlight the clinical implications of the study findings in the Conclusion section without overstating, noting that the results signal that different screening tools may be needed for different sociodemographic identities (e.g, male vs female) or that different cut scores on existing tools may be needed for other categories (e.g., younger vs older age). We’ve added one additional sentence to the Conclusion (line 496-497) noting that we are unaware of prior research on any prognostic tool that explored differential functioning across strata – and signal this as an opportunity for further exploration in this space.**

Conclusion:
Overall, this manuscript represents a well-conducted and timely contribution to the literature on prognostic tools in acute MSK injury and their interaction with sociodemographic factors. The findings are novel, methodologically sound, and the conclusions are appropriately supported by the data. I consider this manuscript suitable for publication.

**Reviewer #2:** This is an exceptionally well-designed and clearly presented study that addresses an important and timely issue in musculoskeletal injury care. The authors chose appropriate, rigorous methods and presented them transparently, with thoughtful attention to prognostic validity and recovery definition. The integration of multiple recovery indicators, the exploration of subgroup effects, and the use of ROC/AUC approaches all contribute meaningful insight to the literature. The manuscript is logically structured, easy to follow, and clinically relevant, and the findings have clear implications for improving risk stratification and patient management in work-related MSK injuries.

I have two minor suggestions.

One is to consider whether any of the dense data presented in table format might be presented graphically in a figure. If this is not practical given the complexity of the data, the manuscript will work as is.

**RESPONSE: This is a well-received comment, and we have indeed attempted different ways to display the variety of results including tabular and graphical formats. In our opinion, having attempted to create several figures and fit them into a single panel, the results are even more difficult to interpret than in the current table format. We’re hopeful the journal typesetter will have some formatting wizardry they can apply to ensure the tables are as interpretable and aesthetically pleasing as possible.**

The second is to consider expanding the discussion point about the finding that high TIDS scores may signal underlying cumulative stress and discrimination that can impede recovery. One potential actionable takeaway might be that clinicians caring for those with a high TIDS score include strategies to identify and address cumulative psychosocial load, not just treat the MSK injury. This may be an interesting avenue for follow-on research.

**RESPONSE: Really nice observation here, we thank the reviewer for that. To acknowledge this, we’ve added the following (lines 398-404): “**This appears to align with prior work showing that the strongest predictors of poor outcome after acute injury are not the magnitude of damage or injury but rather the magnitude of distress experienced.[1, 2] The potential for complex interactions or mutual synergy between prior life stressors and new trauma for predicting future recovery introduces an intriguing direction for further research. One such direction is the effect of integrating trauma-informed/assumed care or other strategies that manage cumulative psychological and social load in supporting recovery after injury, beyond focusing solely on the tissues at fault.”

Overall, this is high-quality work, and I congratulate the authors on a strong contribution to the field. I support acceptance of the manuscript.

**Reviewer #3**: This manuscript addresses an important and underexplored question at the intersection of prognostic research, work-related MSK injury, and social inequities. The dataset and open data sharing are valuable strengths. With revisions to strengthen the statistical rigor and more cautious, clearly exploratory framing of subgroup findings—especially regarding dichotomization, multiple comparisons, power, and generalizability—the study could make a useful contribution to the literature on personalized and equitable prognosis in injured workers.

Major Comments:

1. Overall technical soundness and conclusions:

The prospective cohort design and choice of measures are appropriate, and the main descriptive findings (e.g., older workers reporting higher pain, higher discrimination associated with greater distress, and TIDS/EQ-5D-5L generally outperforming NPRS) are broadly supported by the data. However, several analytic choices reduce statistical rigor and the precision of inferences. In my view, the conclusions—especially about differential prognostic performance by subgroup and implications for cut-scores—should be more clearly framed as exploratory and hypothesis-generating rather than confirmatory.
**RESPONSE: The reviewer has acknowledged our own tensions in the work, and we will address the specifics of those in the detailed comments below. We have attempted to be transparent about the exploratory nature of the study throughout, though we’ve now added another flag to this in the first sentence of the Conclusion section: “The purpose of this hypothesis-generating study was to explore…”**

2. Statistical analysis: dichotomization and collapsed categories:

A central concern is the extensive dichotomization and collapsing of variables:

Age, income, and InDI-M are split at the median into “high/low” groups.

Education is collapsed to “university degree vs. no university degree”.

Race/ethnicity is collapsed to “White-identifying vs. non-White-identifying”.

While this may have been motivated by power considerations for subgroup ROC analyses, dichotomization is known to reduce power, obscure non-linear relationships, and increase the risk of misleading findings. The very broad “non-White” category is particularly problematic in an equity-focused study, as it likely masks heterogeneity among minoritized groups.

Suggestions:

Retain age, income, and InDI-M as continuous predictors in at least some models (e.g., logistic regression for recovery outcomes), and clearly label subgroup analyses as secondary/exploratory.

**RESPONSE: We do not disagree with this sentiment, and for purposes of this paper we’re going to endorse retaining the analyses as are. Our concern at this point is the sheer density of data and analyses, on top of which additional analyses risk obscuring the primary focus which is the comparison of AUCs. We also felt it important to adhere to our analytic plan for this paper, being sensitive to the risk of being accused of a ‘fishing expedition’ with additional analyses. However, we acknowledge the value and intent of this suggestion, and for that purpose have added an additional sentence to the limitations section (line 476-479):** We acknowledge that dichotomizing variables can obscure some (non)-linear effects, and expect that with larger samples there will be opportunities to explore finer gradations in differential effects across levels of constructs like age, income, or education.

Provide more detailed racial/ethnic category frequencies in a table or supplement, even if inferential analyses must use a collapsed variable.

**RESPONSE: While we had been sensitive to the risk of re-identifying those who hold less-common racial identities in our data, we agree that presenting the numbers within each category seems safe and will help illustrate the issue. This has added value in that the categories used were those endorsed by the Canadian Institutes of Health Information (CIHI), which may highlight challenges in collecting these. We’ve added parenthetical acknowledgement of where the categories came from under the Methods section (line 148).**

Strengthen the Discussion’s acknowledgement that these broad categories—especially “non-White”—limit interpretability and do not provide definitive evidence for any specific racialized group.

**RESPONSE: As above, the reviewer has directly observed what has represented perhaps our most difficult set of conversations as a research group on this study. We have weighed everything from removing the entire set of results based on ethnoracial identity, to attempting to identify a smaller set of ‘like’ racialized groups, to retaining the analyses as presented. None of these are perfect – but in weighing our concerns, we felt that removing those analyses, contributing to a sort of persistent ‘erasure’ of race-based analyses in research, was the least desirable. Further, any attempts at collapsing the racial identities into 3 or more subgroups quickly became highly problematic – what racial identities should we assume are ‘OK’ with being grouped with others? Does this in fact fix anything or contribute to the problem. In the end, we’ve elected to retain the analyses as presented, partly to call attention to race-based analyses as an issue of research equity, and partly to highlight that if researchers are interested in doing so, it seems clear (at least in our part of Canada) that concerted efforts to recruit people who hold less-prominent or marginalized racial identities are going to be needed. That said, we’re pleased the reviewer has picked up on this, and are hoping that maybe it opens space for us to add the following – which we have attempted to craft with an eye on academic integrity rather than as a political stance on this issue (lines 387-402): “**This is particularly problematic in the ethnoracial comparisons, where we were forced to collapse the responses into overly broad ‘white’ and ‘non-white’ categories owing to the low frequency of non-white identities in this sample – for purposes of both statistical power and to avoid re-identification of those in smaller categories. While we believe race-based analyses can be valuable when performed with appropriate attention to equity and have included those results here in part as an illustration of the practice, we must acknowledge that the overly-broad categorizations used very likely obscure differential effects for many racial identities. These analyses should not be considered definitive evidence that there is or is not a race-based effect on prognostic tools, rather we have also chosen to present these results in this way in the hopes of opening conversation around equity in research. While calls for sex- and gender-based analyses [1] have contributed to more concerted efforts to recruit women and females into research , there has been less attention paid to recruiting different racialized groups. Perhaps our findings, that did not include special considerations for specifically recruiting non-white participants, will contribute to that conversation.”

3. Multiple comparisons and exploratory nature of subgroup AUCs:

The study includes a large number of hypothesis tests: baseline group comparisons, multiple recovery indicators, and extensive subgroup ROC/AUC analyses (Tables 4–6). There is no formal adjustment for multiple testing, and the implications of this for Type I error are only briefly mentioned. This is important for results that are highlighted in the Discussion, such as higher TIDS scores in those with higher discrimination and sex differences in TIDS performance.

Suggestions:

State explicitly in the Methods that analyses are exploratory and not adjusted for multiple comparisons.
**RESPONSE: This has been explicitly stated in both the first sentence of the Methods and Analysis sections.**

In the Results/Discussion, emphasize that significant subgroup effects (e.g., TIDS × sex) should be interpreted cautiously in light of multiplicity and limited power.

**RESPONSE: We’ve added the following sentence to the Discussion (line 462-464):** We also acknowledge the large number of analyses for which the significance threshold (p) was not adjusted, raising the possibility that this is an alpha error.

If feasible, consider a simple sensitivity analysis (e.g., FDR) for key sets of tests and comment on whether main conclusions are robust.

**RESPONSE: We might leave this to the discretion of the editor. We’re happy to report the findings – for context, logistic regression using Sex, TIDS, and TIDS x Sex variables on the work status recovery outcome continued to support the differential effects, as the interaction term was the only one of the 3 predictors retained in the model (OR = 1.07, 95%CI 1.04 to 1.11). Again, we’re sensitive to adding more density to an already dense paper, so we’ll follow the editor’s lead on this one.**

4. Power and precision of subgroup analyses:

Although you exclude subgroup ROC analyses with very small cells, many of the reported subgroup AUCs still have wide confidence intervals and non-significant between-group Z-tests. At times the Discussion appears to give qualitative emphasis to differences that are statistically non-significant and imprecise.

Please make clearer in the text that these subgroup comparisons are underpowered and that the study was not designed to definitively test interaction effects (e.g., sex × tool, age × tool). Where specific differences are highlighted (such as better TIDS performance in females), present them explicitly as hypotheses for future, larger studies rather than firm conclusions.

**RESPONSE: We’ve gone through the Discussion to try to make more clear that these effects are ‘potential’ or require further confirmation. This was added once in Paragraph 2 and twice Paragraph 7 of the Discussion.**

5. Recovery definitions and discrepancies:

Using three distinct recovery indicators (self-rated global recovery, self-reported work status, and administrative discharge status) is a major strength and reveals substantial differences in “recovery” rates depending on how recovery is defined. This deserves more explicit discussion.

I encourage you to further reflect on:

How administrative “full recovery” (driven by clinician/funder judgement) may be influenced by non-clinical factors.

What it means for prognostic validation when patient self-report and administrative outcomes diverge (i.e., which outcome should a “good” prognostic tool prioritise?).

**RESPONSE: We resonate highly with the reviewer on this item – indeed reflecting on the meaning of ‘recovery’ in the context of physical rehabilitation has been a part of the lead author’s research program for 15 years (though clearly he has yet to crack it). We hope the publication of this manuscript will encourage all readers to reflect upon what they mean by the notion of recovery, and will continue to push this field forward.**

Clarifying in the Methods how ambiguous administrative cases were coded would also be helpful.

6. Generalizability and context:

The study is conducted in a single jurisdiction, provider network, and funding scheme (WSIB PoC in Ontario). This context likely limits generalizability to other compensation systems, uninsured or informally employed workers, and different health systems. Although some of this is acknowledged, I recommend slightly strengthening the Discussion to more clearly state these external validity limitations.

**RESPONSE: Good catch, we missed that one. We’ve now added the following to the Limitation paragraph: “**This work was conducted within a single Canadian province, with rehabilitation provided by clinics within a single large network, and under the funding models and guided by protocols of a single rehabilitation funder. While these helped strengthen some aspects of internal validity, they also provide questions on generalizability that would benefit from work outside of these jurisdictions.”

7. Conflict of interest and tool ownership:

The competing interests statement is transparent about the developer of the TIDS being an author and about the tool’s open license. Given that TIDS is one of the focal tools and generally performs well, a brief reminder of this relationship in the Methods or Discussion, coupled with a note that analyses followed pre-specified methods and that raw data are publicly available, would further reinforce transparency.

**RESPONSE: If we can add a comment to the disclosure statement indicating that raw data are available we’re happy to do so. We also noted on this review that we neglected to include the clinicaltrials.gov registration number within the manuscript itself, which made clear the variables and tools that were being used in this series of studies. We’ve now added that under the Methods section.**

Minor Comments:

1. Study period:

The Results report recruitment as “From October 2023 to May 2025”, which appears to be a typographical or dating error. Please verify and correct the study dates.

**RESPONSE: This is actually the correct timeframe. This study is part of a larger set of projects all funded under the same grant. Recruitment and completing all follow up took about 17 months all total.**

2. “Cross-sectional and longitudinal” wording:

It may help readers if you explicitly state early in the Methods that the design involves a baseline assessment with an 8-week follow-up, clarifying that the longitudinal component is relatively short.
**RESPONSE: We’ve added the explicit comment about the short 8-week follow up to the first sentence of the Methods.**

3. Language and readability:

The manuscript is clearly written and in standard English. A light edit at revision could shorten some long sentences in the Discussion and check for minor typographical and numerical inconsistencies.
**RESPONSE: Through the process of this revision we’ve identified some lengthy sentences and have separated those. If we see any more during the proofing stage we’ll address them at that time.**

4. Tables 4–6:

These tables are dense. Consider improving readability by clearly marking which AUCs are significantly >0.5 and which between-group differences are significant, and by visually highlighting only those subgroup effects you intend to emphasize in the text.

**RESPONSE: We hope the bolded fonts have come through clearly on the tables. We’ve attempted to add table footers to explain where the key differences are (e.g., sex x TIDS for self-rated work outcome). They are indeed dense tables, though we’re hopeful that once formatted to journal standards they will look better. They may need to be vertically oriented in print.**

6. PLOS authors have the option to publish the peer review history of their article ([what does this mean?](https://track.editorialmanager.com/CL0/https:%2F%2Fjournals.plos.org%2Fplosone%2Fs%2Feditorial-and-peer-review-process%23loc-peer-review-history/1/010f019adca3c725-40e1cc15-dfcd-490e-a28b-6155a29b964b-000000/AbDFrZ4gr_XSIqQFCU8AlZWm-B3qJlbkpNwQSrkwLcw=239)). If published, this will include your full peer review and any attached files.

If you choose “no”, your identity will remain anonymous but your review may still be made public.

**Do you want your identity to be public for this peer review?** For information about this choice, including consent withdrawal, please see our [Privacy Policy](https://track.editorialmanager.com/CL0/https:%2F%2Fwww.plos.org%2Fprivacy-policy/1/010f019adca3c725-40e1cc15-dfcd-490e-a28b-6155a29b964b-000000/ez2NA3jskGKApnqs1WOiSA-hvdGfiWqzssfI831fzqg=239).

Reviewer #1: **Yes:** Mohammad Fayaz

Reviewer #2: No

Reviewer #3: No
